# Protocol for pyrotinib cardiac safety in patients with HER2-positive early or locally advanced breast cancer–The EARLY-MYO-BC study

**DOI:** 10.3389/fcvm.2023.1021937

**Published:** 2023-02-10

**Authors:** Yezi Chai, Meng Jiang, Yaohui Wang, Qiming Liu, Qifan Lu, Zhengyu Tao, Qizhen Wu, Wenjin Yin, Jinsong Lu, Jun Pu

**Affiliations:** ^1^Division of Cardiology, State Key Laboratory for Oncogenes and Related Genes, Key Laboratory of Coronary Heart Disease, School of Medicine, Renji Hospital, Shanghai Jiao Tong University, Shanghai, China; ^2^Department of Breast Surgery, School of Medicine, Renji Hospital, Shanghai Jiao Tong University, Shanghai, China

**Keywords:** pyrotinib, cardiotoxicity, HER2-positive breast cancer, therapy-related cardiac dysfunction, echocardiography, magnetic resonance

## Abstract

**Background and aim:**

Cardiotoxicity has become the most common cause of non-cancer death among breast cancer patients. Pyrotinib, a tyrosine kinase inhibitor targeting HER2, has been successfully used to treat breast cancer patients but has also resulted in less well-understood cardiotoxicity. This prospective, controlled, open-label, observational trial was designed to characterize pyrotinib’s cardiac impacts in the neoadjuvant setting for patients with HER2-positive early or locally advanced breast cancer.

**Patients and methods:**

The EARLY-MYO-BC study will prospectively enroll HER2-positive breast cancer patients who are scheduled to receive four cycles of neoadjuvant therapy with pyrotinib or pertuzumab added to trastuzumab before radical breast cancer surgery. Patients will undergo comprehensive cardiac assessment before and after neoadjuvant therapy, including laboratory measures, electrocardiography, transthoracic echocardiography, cardiopulmonary exercise testing (CPET), and cardiac magnetic resonance (CMR). To test the non-inferiority of pyrotinib plus trastuzumab therapy to pertuzumab plus trastuzumab therapy in terms of cardiac safety, the primary endpoint will be assessed by the relative change in global longitudinal strain from baseline to completion of neoadjuvant therapy by echocardiography. The secondary endpoints include myocardial diffuse fibrosis (by T1-derived extracellular volume), myocardial edema (by T2 mapping), cardiac volumetric assessment by CMR, diastolic function (by left ventricular volume, left atrial volume, E/A, and E/E’) by echocardiography, and exercise capacity by CPET.

**Discussion:**

This study will comprehensively assess the impacts of pyrotinib on myocardial structural, function, and tissue characteristics, and, furthermore, will determine whether pyrotinib plus trastuzumab is a reasonable dual HER2 blockade regimen with regard to cardiac safety. Results may provide information in selecting an appropriate anti-HER2 treatment for HER2-positive breast cancer.

**Clinical trial registration:**

https://clinicaltrials.gov/, identifier NCT04510532

## 1. Introduction

Breast cancer is the most commonly diagnosed tumor and is also the most malignant female tumor (1). Advances in pharmacotherapies have resulted in tremendous progress in breast cancer treatment (2). However, survival benefits are increasingly offset by adverse drug reactions, particularly cardiotoxicity. Cardiotoxicity can result in cancer therapy-related cardiac dysfunction (CTRCD), myocarditis, arrythmias, and other adverse outcomes (3, 4). Remarkably, cardiotoxicity may occur during various stages of cancer, at different ages, and even in patients who are considered as theoretically low risk at baseline (5–8). Hence, anticancer drugs with less cardiotoxicity are urgently needed.

HER2-directed drugs have become a cornerstone for the treatment of HER2-positive breast cancer, and they have shown less cardiotoxicity than traditional anthracyclines (2). Currently, dual HER2 blockade with trastuzumab and pertuzumab is recommended by the National Comprehensive Cancer Network (NCCN) guidelines as an important neoadjuvant therapy component for patients with early or locally advanced HER2-positive breast cancer (9). However, studies have recently shown that this regimen has a higher incidence of clinical heart failure than trastuzumab alone (10).

Pyrotinib is a small-molecule tyrosine kinase inhibitor (TKI) that simultaneously inhibits HER2 homodimerization and heterodimerization. After demonstrating significant anticancer activity in the PHOEBE trial and other clinical trials, the National Medical Products Administration of China approved pyrotinib in 2020 for the treatment of HER2-positive advanced or metastatic breast cancer (11). As a promising HER2-targeted agent, adding pyrotinib to trastuzumab and neoadjuvant chemotherapy further improved the pathological complete response (pCR) rate up to 69.81% in patients with early or locally advanced breast cancer in a phase II NeoATP trial (12). To date, the adverse drug reactions of pyrotinib include adverse gastrointestinal reactions and hand-foot syndrome. Unfortunately, no research has yet evaluated its cardiovascular impacts.

Cardiac imaging, including echocardiography and cardiac magnetic resonance (CMR), is critical for cardiac assessment in breast cancer patients. Of the cardiac functional parameters, left ventricular ejection fraction (LVEF) and global longitudinal strain (GLS) derived from echocardiography are the most widely used to define CTRCD. Up to 39.1% of breast cancer patients exposed to trastuzumab and anthracycline presented with CTRCD during treatment or follow-up (13). Furthermore, CMR, the non-invasive reference technique for myocardial volumetric and functional assessment, is also capable of offering additional information on cardiac tissue properties. Using CMR, increased intracellular matrix can be detected by T1-derived extracellular volume (ECV) quantification (14), and myocardial edema can be identified on CMR T2-mapping images (15, 16). Hence, multi-modality cardiac imaging can enable a comprehensive picture of myocardial features after anti-cancer therapy.

Here, we registered the EARLY-MYO-BC study in order to describe pyrotinib’s cardiac impacts and to further determine whether novel dual HER2 blockade using pyrotinib plus trastuzumab is non-inferior to the NCCN-recommended regimen (pertuzumab plus trastuzumab) with regard to cardiac safety during the course of neoadjuvant treatment.

## 2. Methods

### 2.1. Study overview

The EARLY-MYO-BC (Early Assessment of Myocardial Injury in Patients Receiving Neoadjuvant Pyrotinib Therapy with Early or Locally Advanced HER2-Positive Breast Cancer) is a prospective, open-label, observational study (ClinicalTrials.gov identifier NCT04510532) that will enroll patients with early or locally advanced HER2-positive breast cancer from the Department of Breast Surgery at the Renji Hospital for cardiac assessment from the initiation of neoadjuvant pyrotinib or pertuzumab therapy. The registry has been approved by the local Ethics Committee of Renji Hospital. All patients will provide fully informed written consent. The study protocol conforms with the principles of the 2013 Declaration of Helsinki and the International Conference on Harmonization Good Clinical Practice Guideline.

### 2.2. Study population

#### 2.2.1. Eligibility and exclusion criteria

Patients are eligible for this trial if they meet all of the following criteria: (1) female patients between 18 and 70 years of age; (2) newly diagnosed and histologically confirmed early or locally advanced HER2-positive invasive breast cancer (Stage IIA-IIIC); (3) no signs of cardiac discomfort; (4) normal range of baseline cardiac biomarkers [troponin I (TNI), brain natriuretic peptide (BNP), N-terminal pro-brain natriuretic peptide (NT-proBNP)], electrocardiography (ECG), and baseline LVEF ≥ 50% from echocardiography; (5) able to complete face-to-face follow-up visits during neoadjuvant therapy; (6) provide signed written informed consent.

The exclusion criteria are as follows: (1) metastatic breast cancer confirmed histologically or by imaging tests; (2) prior history of medical anticancer treatments; (3) documented coronary heart disease or cardiomyopathy; (4) angina pectoris requiring medication; (5) clinically significant valvular disease; (6) unstable arrhythmias; (7) uncontrolled blood pressure; (8) severe chronic or acute renal failure (glomerular filtration rate < 30 ml/min/1.73 m^2^); (9) severe liver diseases; (10) prior history of immunodeficiency or organ transplantation; (11) pregnant or lactating; (12) unable to take or absorb tablets; (13) contraindications to CMR; (14) any other medical condition assessed by the investigator as inappropriate to participate.

#### 2.2.2. Informed consent

A trained researcher will be responsible for providing all necessary information about this study to the potential participants. It will be made clear to the participants that they are under no obligation to take part in the trial, and their usual care will not be affected by their decision. All participants can withdraw their consent during the trial without providing a reason or explanation. They will be given a sheet with contact details for the research team and instructions on what to do if they wish to withdraw or require further information.

### 2.3. Groups and treatment procedure

**FIGURE 1:**
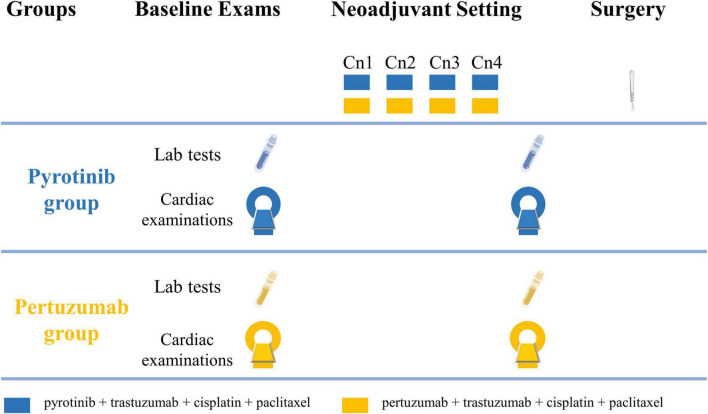
Study flowchart. Cardiac examinations include electrocardiography, echocardiography, cardiac magnetic resonance, and cardiopulmonary exercise testing. Cn, neoadjuvant cycle.

The study flowchart is shown in Figure 1. Cardiac assessments are scheduled to be conducted at baseline and before radical breast cancer surgery. Four cycles of neoadjuvant therapy will be given before radical breast cancer surgery and each cycle will last for 28 days.

Pyrotinib group treatment regimen: Cisplatin (25 mg/m^2^ at day 1, 8, 15), paclitaxel (80 mg/m^2^ at day 1, 8, 15, 22), and trastuzumab (4 mg/kg at loading dose, then 2 mg/kg per week) will be administered intravenously to patients in the ambulatory chemotherapy ward. Patients will take 400 mg pyrotinib orally once per day.

Pertuzumab group treatment regimen: Cisplatin, paclitaxel, and trastuzumab will be administered to patients with the same regimen as the pyrotinib group. Pertuzumab will be intravenously given at an 840 mg loading dose, followed by 420 mg every 3 weeks in the ambulatory chemotherapy ward.

To obtain baseline normal myocardial values for comparison, age- and gender-matched healthy volunteers without cardiovascular diseases (CVDs) will be recruited as negative controls.

### 2.4. Cardiac assessment

#### 2.4.1. Clinical assessment

Demographic features including age and body mass index (BMI) will be prospectively collected at baseline, as well as blood pressure, heart rate, risk factors for cardiovascular events (e.g., hypertension, hyperlipidemia, diabetes, current smoking, and family history of early onset CVD), tumor location, tumor stage, and history of cancer and other diseases. Baseline cardiac risk assessment will be performed using the statement-recommended algorithm (17).

#### 2.4.2. Laboratory measurements

Laboratory measurements will include: (1) cardiac markers (TNI, BNP, NT-proBNP, and soluble ST2); (2) complete blood counts (white blood cell counts, hemoglobin, hematocrit, and platelet counts); (3) hepatic function markers (alanine aminotransferase, aspartate aminotransferase, and total bilirubin); (4) renal function markers (creatinine and blood urea nitrogen); (5) lipid profile (triglycerides, total cholesterol, high-density lipoprotein, and low-density lipoprotein); (6) fasting glucose; (7) inflammatory markers (C-reactive protein, interleukin-1β, interleukin-6, interleukin-8, and tumor necrosis factor-α).

#### 2.4.3. Cardiac examination

ECG, echocardiography, cardiopulmonary exercise testing (CPET), and CMR data will be recorded in the study database.

##### 2.4.3.1. Electrocardiography and echocardiography image acquisition

A resting 12-lead ECG will be used following the current guideline-recommended standard protocols to record the cardiac electrophysiology of breast cancer patients (18).

Echocardiography will be performed to obtain left ventricular (LV) morphology and function (Vivid E95 ultrasound system, GE Vingmed Ultrasound, Horten, Norway) following the standard recommendation (19). Two level 3 sonographers (who are blinded to the results of the other examinations) will analyze the images separately. Echocardiographic measurements include left atrial (LA) dimension, LV end-diastolic and end-systolic dimensions, LV septal and posterior wall thicknesses, and LVEF. Myocardial strain [global circumferential strain (GCS), GLS and global radial strain (GRS)] will be used to assess tissue deformation.

Diastolic function will be assessed by the ratio between early (E) and atrial (A) peak diastolic velocities of LV inflow (E/A), the ratio between E and peak early (E’) tissue Doppler of the mitral annulus (E/E’), and the LA dimension. Within 24 h of the ECG and echocardiographic examination, a CMR scan and CPET will be scheduled.

##### 2.4.3.2. Cardiopulmonary exercise testing

CPET will be conducted to evaluate the cardiac-pulmonary function of breast cancer patients by continuously measuring breath-by-breath expired gases before, during, and after exercise. Testing will start with 3-min unloaded cycling and gradually achieve peak exercise using the 10 Watt/minute incremental ramp protocol. Patients are encouraged to maintain exercise until exhaustion. The test will be considered valid with a perceived exertion rating >18 on the Borg scale or a respiratory exchange ratio > 1.1 (20–22). A 12-lead ECG and blood pressure measurement will be recorded every 3 min. The typical CPET variables include peak oxygen uptake, peak metabolic equivalent, ventilatory efficiency, oxygen pulse, peak blood pressure, and anaerobic threshold.

##### 2.4.3.3. CMR image acquisition

CMR will be performed using a Prisma 3.0-Tesla magnetic resonance (MR) scanner (Siemens Healthineers, Germany). The imaging protocol is consistent with the statement from the Society for Cardiovascular Magnetic Resonance (23). The full imaging protocol will include standard cine imaging for cardiac morphological and functional assessment, T1 mapping and late gadolinium enhancement (LGE) for myocardial fibrosis assessment, and T2 mapping for myocardial edema assessment. Two cardiologists (with >8 years of experience in CMR and blinded to the clinical information) are responsible for image post-processing using CVI42 5.13.9 software (Circle Cardiovascular Imaging, Calgary, Canada).

Cine imaging: Standard cine images will be acquired with end-expiratory breath hold using retrospectively gated, balanced, steady-state, free precession sequences in three long-axis planes and sequential short-axis slices from the atrioventricular ring to the LV apex. The typical cine sequence parameters are as follows: field of view (FOV), 360 × 360 mm; repetition time (TR), 3.39 ms; echo time (TE), 1.49 ms; flip angle (FA), 58°; voxel size, 1.4 × 1.4 × 8.0 mm^3^. The LV endocardial and epicardial borders are end-diastole and end-systole contoured to accomplish cardiac volumetric and strain analysis. All volumetric indices and mass will be indexed to body surface area. Trabeculations and papillary muscles will be included in LV mass calculations and excluded from LV volumes. Myocardial strain will also be assessed. GCS and GRS will be obtained in the short-axis view at the apical, midventricular, and basal levels, and GLS will be obtained in the long-axis view (2-, 3-, and 4-chamber images).

T2 mapping: T2 mapping will be performed with a T2-prepared, single-shot, multi-echo, steady-state free precession (SSFP) technique, and three T2P-SSFP images will be obtained in the short axis at the basal, mid, and apical levels. Typical sequence parameters are as follows: FOV, 360 × 360 mm; TR, 3.15 ms; TE, 1.38 ms; FA, 12°; matrix, 224 × 160; voxel size, 1.6 × 1.6 × 8.0 mm^3^, bandwidth, 1175 Hz/Px.

Pre- and post-contrast T1 mapping: A SSFP, single breath-hold, modified Look-Locker inversion recovery sequence will be used for T1 mapping before and 15 min after contrast application. Three slices will be obtained for pre- and post-contrast T1 mapping in the short axis at the basal, mid, and apex. Typical parameters are as follows: FOV, 360 × 360 mm; TR, 2.44 ms; TE, 1.12 ms; FA 35°; voxel size, 1.4 × 1.4 × 8.0 mm^3^; 160 phase-encoding steps; sensitivity encoding factor = 2. Regions of interest will be placed on the entire myocardium to obtain T1 values, but will exclude the blood pool, papillary muscles, chordae, and trabeculations. To detect interstitial matrix changes ECV is calculated based on the combination of pre- and post-contrast T1 values with the formula: ECV = (ΔR1myocardium/ΔR1blood) • (1 - hematocrit) where R1 = 1/T1 time (24).

LGE: A LGE scan will be performed 10 min after intravenous administration of gadolinium contrast and will employ a phase-sensitive reconstruction (PSIR) covering the entire left ventricle from basal to apex in short-axis views. Typical parameters are as follows: TR, 3.87 ms; TE, 1.52 ms; FA, 20°; voxel size, 0.8 × 0.8 × 8.0 mm^3^. LGE positive is defined as hyperenhancement on the myocardium (>5 × S.D. of reference region).

### 2.5. Study endpoint

To determine whether pyrotinib is non-inferior to pertuzumab with regard to cardiac safety when combined with trastuzumab during the course of neoadjuvant therapy, our primary endpoint is designed to compare the relative change in GLS between the two groups by echocardiography. The secondary endpoints are assessed by CMR and CPET including the relative changes in (1) myocardial diffuse fibrosis (by T1-derived ECV), (2) myocardial edema (by T2 mapping), (3) myocardial volumetric and diastolic function (by LV volume, LA volume, E/A, E/E’) and (4) exercise capacity between the two groups.

### 2.6. Reproducibility

One-fourth of the patients will be randomly chosen for imaging measurement reproducibility assessment 1 month after the first measurement. The interobserver and intraobserver imaging measurement reproducibility will be assessed by intraclass correlation analyses.

### 2.7. Sample size calculation

The sample size calculation is based on our preliminary research. The mean relative changes in GLS before and after treatment of 10 patients receiving pyrotinib and 10 patients receiving pertuzumab were 6.15 and 5.00 with standard deviations (SDs) of 8.10 and 7.73, respectively. Assuming a non-inferiority margin of 3.8, a sample ratio (n pyrotinib/n pertuzumab) of 1, a type I error (one-sided) of 5%, a power of 80%, and a missing rate of 10%, a final sample size of at least 61 subjects in each group will be required to detect the non-inferiority of pyrotinib to pertuzumab with regard to cardiac safety.

### 2.8. Statistical analysis

Data will be tested for normal distribution by the Kolmogorov–Smirnov test. Normally distributed data are expressed as mean ± SD. Non-normally distributed data are expressed by median and interquartile range (IQR). Categorical data are expressed as frequencies and percentages. Non-inferiority will be assessed on the primary endpoint to compare the differences between the two groups in terms of relative changes in GLS from baseline to completion of neoadjuvant therapy. Baseline characteristics and secondary endpoints will be analyzed using Student’s *t*-test or Mann–Whitney U test for continuous variables that are normally or non-normally distributed, respectively, or using chi-square test or Fisher exact test for categorical data. Logistic regression will be employed to identify the predictive value of baseline clinical features (age, BMI, prior history of hypertension and hyperlipidemia, etc.), biomarkers (TNI, BNP, NT-proBNP, ST2, etc.), and imaging indicators in CTRCD development. The interobserver reliability will be evaluated by intraclass correlation coefficient (ICC) for continuous variable and Kappa value for categorical variable. Except for the non-inferiority test of the primary endpoint, all reported *P*-values are two-sided; those under 0.05 are considered to be statistically significant. Analyses are performed using SPSS 26.0 and R software (version 3.4.1).

## 3. Discussion

To the best of our knowledge, the EARLY-MYO-BC registry is the first to explore pyrotinib’s cardiac impacts in the neoadjuvant setting for patients with early or locally advanced HER2-positive breast cancer.

Over the past decades, tremendous advances have been made in pharmacotherapies for breast cancer, especially in targeted therapy, reducing the 10-year risk of dying from breast cancer by 6.4% in patients diagnosed with early HER2-positive breast cancer (2, 25). These advances in anti-cancer therapies, however, came with an increased frequency of cardiotoxic events that must be addressed. Survivors were revealed to have approximately 2-fold higher risk of CVD-related mortality than the general population 8 years after breast cancer diagnosis (26). Cancer therapy-related cardiotoxicity might exacerbate underlying heart disease or initiate *de novo* cardiac problems. Furthermore, cardiotoxicity can complicate and even cause interruption or discontinuation of cancer therapy, which significantly reduces breast cancer patients’ survival benefit (27, 28). Indeed, up to 11% of early breast cancer patients exposed to trastuzumab experienced an interruption of therapy due to cardiotoxicity (28). Therefore, early identification of anticancer drug-related cardiotoxicity characteristics and severity is extremely valuable in rationally selecting treatment strategies to avoid severe cardiac injury and cancer treatment discontinuity.

The optimal breast cancer therapy is driven by molecular subtype. For HER2-positive breast cancer, anti-HER2 therapy, especially dual HER2 blockade (trastuzumab and pertuzumab), has been recommended as part of the preferred neoadjuvant therapy by the NCCN (9). Notably, the incidence of cardiotoxicity varies widely among anti-HER2 drugs depending on the nature of the drugs, therapy duration, and underlying patient comorbidities. CTRCD has been described in 3–10% of early breast cancer patients treated with trastuzumab and in 19% treated with trastuzumab plus anthracyclines (2, 29). The mechanism of trastuzumab-induced cardiotoxicity involves inhibition of the neuregulin-1/ErbB signaling pathway, which disequilibrates cardiomyocyte homeostasis and leads to myocardial injury (30, 31). Other anti-HER2 drugs, such as lapatinib, resulted in a lower incidence of CTRCD than trastuzumab (2–9% with lapatinib), probably due to their distinct binding epitopes on the extracellular domain of the HER2 receptor (30, 32–34). Thus, anti-HER2 therapies have different mechanisms and incidences of cardiac injury. For dual HER2 blockade, the cardiotoxicity might be synergistic. Specifically, dual HER2 blockade combining trastuzumab and pertuzumab resulted in a higher incidence of heart failure compared with trastuzumab alone (10, 35). Hence, it is imminent to identify an optimal combination of HER2-directed agents that balance efficacy and cardiotoxicity.

Pyrotinib is an oral small-molecule TKI of multiple HER family members, enabling concomitant blockade of HER2 homodimerization and heterodimerization. In patients with HER2-positive relapsed or metastatic breast cancer, pyrotinib was shown to prolong the median progression-free survival more significantly than lapatinib (12.5 months vs 6.8 months) (11). As a novel dual HER2 blockade regimen in the neoadjuvant setting, pyrotinib combined with trastuzumab demonstrated potent antitumor activity with a pCR rate of 55.1–69.81%, higher than the pCR rate of 54.0% obtained with trastuzumab combined with pertuzumab (12, 36, 37). However, the cardiotoxicity of pyrotinib has rarely been studied in single or dual HER2 blockade regimens. We propose that if the combination of trastuzumab and pyrotinib shows non-inferiority to the combination of trastuzumab and pertuzumab with regard to cardiac safety, the former regimen might serve as an alternative option for dual HER2 blockade in the neoadjuvant setting.

To date, assessment of cancer therapy-related cardiotoxicity has relied largely on serum biomarker measures (TNI, BNP, NT-proBNP, etc.) and cardiac imaging tests (echocardiography, CMR, etc.) (17, 38, 39). As the most commonly used cardiac biomarkers, TNI and BNP play a limited role in detecting early subclinical cardiac impairment since biomarker abnormality usually suggests a present or irreversible myocardial injury (40, 41). Although NT-proBNP has been shown to predict future heart failure in the general population, evidence-based data for NT-proBNP regarding the detection of cancer therapy-related cardiotoxicity is still lacking (42–47). Clinically, LVEF is the mainstay of imaging metrics for cardiac function evaluation. However, relying solely on LVEF assessment to identify cardiotoxicity, especially in those presenting with early subclinical cardiac dysfunction, is far from adequate. GLS, an emerging imaging metric, provides quantitative assessment for the early detection of pre-clinical heart failure in cancer patients and has been recommended by the Cardio-Oncology Council of the European Society of Cardiology (17). GLS reduction helps to predict subsequent cardiotoxicity in cancer patients before the decrease of LVEF (48–50). We therefore selected GLS change during neoadjuvant therapy as our primary endpoint. Furthermore, previous literature revealed that the increase in chemotherapy-induced myocardial fibrosis (indicated by T1-derived ECV) and edema (indicated by T2 mapping) on CMR images implied cardiotoxicity when LVEF was still within normal range (14–16). With this in mind, the secondary endpoints in our study further explore the myocardial tissue alterations in patients treated with pyrotinib or pertuzumab.

In conclusion, this study aims to comprehensively assess pyrotinib’s cardiac impacts and, further, to determine whether pyrotinib plus trastuzumab is non-inferior to pertuzumab plus trastuzumab with regard to cardiac safety. The myocardial structure, function, and tissue characteristics will be explored, and the findings will provide valuable information for oncologists with respect to selecting appropriate treatment options for patients with breast cancer.

## Ethics statement

The studies involving human participants were reviewed and approved by the local Ethics Committee of Renji Hospital. The patients/participants provided their written informed consent to participate in this study.

## Author contributions

YC and YW: design, patient recruitment, data acquisition and analysis, manuscript drafting and revision, and approval of the final version of the manuscript. QML, QFL, ZT, and QW: data acquisition. MJ, WY, JL, and JP: study concept and design, study supervision, drafting, revision, and approval of the final version of the manuscript. All authors contributed to the article and approved the submitted version.
